# Embryonal tumor with multilayered rosettes in a teenager

**DOI:** 10.4322/acr.2021.373

**Published:** 2022-04-14

**Authors:** Kofi Ulzen-Appiah, Kafui Patrick Akakpo

**Affiliations:** 1 Cape Coast Teaching Hospital, Department of Pathology, Cape Coast, Ghana

**Keywords:** Brain neoplasms, Neuroectodermal Tumors, Primitive, Autopsy, Cerebellar Neoplasms

## Abstract

Background: Embryonal tumor with multilayered rosettes (ETMR), NOS/C19MC- altered, is a rare and recently classified highly aggressive malignant brain tumor in the 2021 World Health Organization (WHO) classification of tumors of the central nervous system 5^th^ edition. They are mostly diagnosed in children before the age of three years. Most of them are located in the supratentorial region. Prior to the reclassification of ETMR as a single entity, three distinct tumors, namely, embryonal tumor with abundant neuropil and true rosettes (ETANTR), ependymoblastoma (EBL) and medulloepithelioma (MEPL) were recognized. Recent studies showed that all the three entities have multilayered rosettes on morphology, sharing a common amplification of the C19MC locus at the chromosome 19q13.42 by fluorescence in situ hybridization, and highly specific immunohistochemical staining for LIN28A rendered their reclassification as a single entity. Report: A 13-year-old girl was rushed to the emergency room unconscious, with no return of spontaneous circulation after cardiopulmonary resuscitation. Autopsy revealed a left cerebellar hemisphere hemorrhagic tumor which histopathological examination revealed a multilayered ependymoblastic rosettes with abundant neuropil. The multilayered rosettes showed reactivity for vimentin but non-reactivity for pan-cytokeratin, the zones with abundant neuropil were reactive for synaptophysin consistent with a diagnosis of embryonal tumor with abundant neuropil and true rosettes now ETMR, NOS (WHO Grade 4) due to the lack of genetic testing for amplification of C19MC. Conclusion: ETMR is a highly aggressive CNS embryonal tumor with extremely poor prognosis. It should be considered in the differential diagnosis of pediatric brain tumors. Multilayered rosettes are a useful clue to histologic diagnosis.

## INTRODUCTION

Embryonal tumor with multilayered rosettes (ETMR), C19MC altered is a recently reclassified tumor entity in the recent update of the 2021 World Health Organization (WHO) classification of tumors of the central nervous system 5^th^ edition.^1^ It consists of a group of three morphologically different embryonal tumors, which were characterized as distinct entities in the 2007 fourth edition of the WHO blue book.^2^ These include embryonal tumor with abundant neuropil and true rosettes (ETANTR), ependymoblastoma (EBL) and medulloepithelioma (MEPL). The basis for unifying these distinct embryonal tumors is their expression of a common molecular signature, which is C19MC amplification and highly specific immunohistochemical staining for LIN28A.^3^ Morphologically, the majority of the tumors share the presence of multilayered rosettes, which express reactivity for vimentin and LIN28A and negative reactivity for pan-cytokeratin.^4^ They correspond histologically to WHO grade 4 embryonal tumors.^5^

ETMRs usually affects children less than 4 years old and may occur in any part of the brain, although they are typically seen in the supratentorial region.^6^ Patients usually present with signs and symptoms of raised intracranial pressure and/or neurologic deficits.^4^ On magnetic resonance imaging (MRI), they show cystic components and intratumoral hemorrhage.^7^

The treatment approach has not been well defined due to its rarity, but a common multimodality is maximal safe surgical resection, age and risk adapted radiotherapy and chemotherapy.^8^ The behavior of this WHO grade 4 tumor is largely aggressive and the prognosis is dismal.^3^

We report a case of a thirteen-year-old female who died suddenly. Postmortem examination revealed an infratentorial left cerebellar hemorrhagic tumor. Histopathological examination showed features consistent with the previous embryonal tumor known as embryonal tumor with abundant neuropil and true rosettes (ETANTR) now reclassified as embryonal tumor with multilayered rosettes, not otherwise specified (ETMR, NOS) due to our inability to perform fluorescence in situ hybridization (FISH) studies to detect amplification of C19MC.

## CASE REPORT

A 13 -year-old female was rushed to the accident and emergency department of our facility in an unconscious state. According to her caregivers, she fell and hit the head on the ground 4 days prior, but was well till her current state of unconsciousness. Cardiopulmonary resuscitation was done but no return of spontaneous circulation was achieved and she was declared dead by the attending clinician. A coroner’s autopsy was requested thereof.

**Figure 1:**
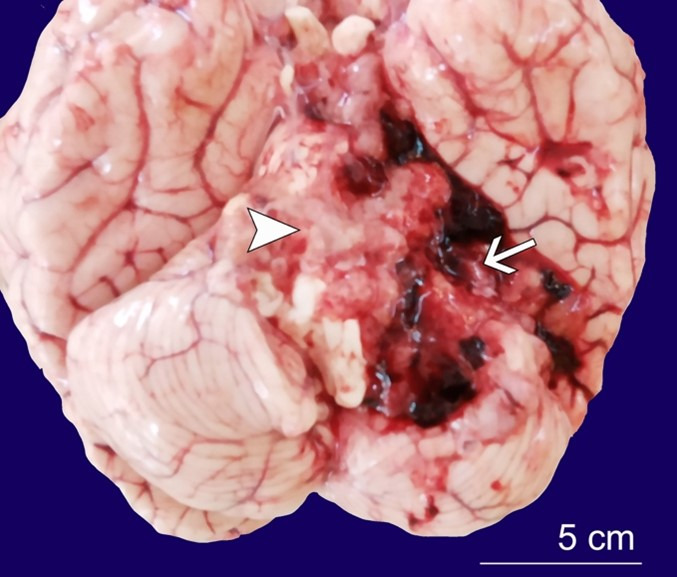
Brain macroscopic view shows the base of brain with (infratentorial) left cerebellar tumor with surrounding hemorrhage (arrow) and liquefactive necrosis (arrowhead).

Gross findings of the brain showed cerebral edema evidenced by increased weight (1,500g) (reference range 1,200-1400g), as well as flattening of the gyri and narrowed sulci. Noted at the base of the brain was a partly necrotic tumor with surrounding haemorrhage of the left cerebellar hemisphere measuring 60x60mm (Figure 1).

**Figure 2:**
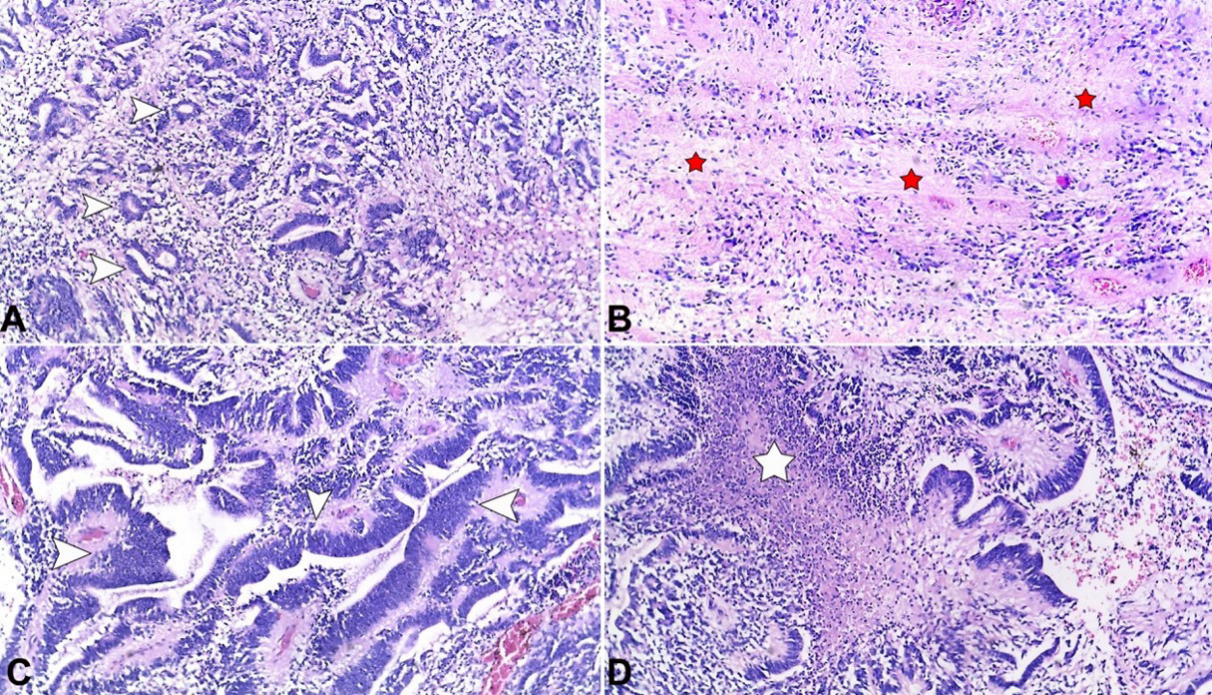
Photomicrographs of the tumor – **A –** shows biphasic tumor cellular primitive cells arranged in ependymoblastic rosettes - (white arrowheads), **C** – papillae and trabeculae (white arrowheads) with **B** – intervening hypocellular zones composed of differentiated neuropil and sheets of primitive cells (red stars); **D –** Areas of necrosis (white star). (**A – D**, H&E x 100)

Microscopic sections of the tumor showed a biphasic hyper-cellular and hypo-cellular tumor. The cellular areas composed of primitive cells arranged in sheets, papillae, tubules (reminiscent of immature neural tubes), and multilayered ependymoblastic rosettes (with central lumen) (Figures 2A &2C) and intervening hypo-cellular zones with abundant neuropil containing occasional true rosettes (Figure 2B). The primitive cells show increased nuclear to cytoplasmic ratio and round to oval nuclei. Mitotic figures are abundant. Necrosis was seen in cellular zones (Figure 2D). In areas there are microcalcifications and the tumor invades the adjacent parenchyma.

**Figure 3:**
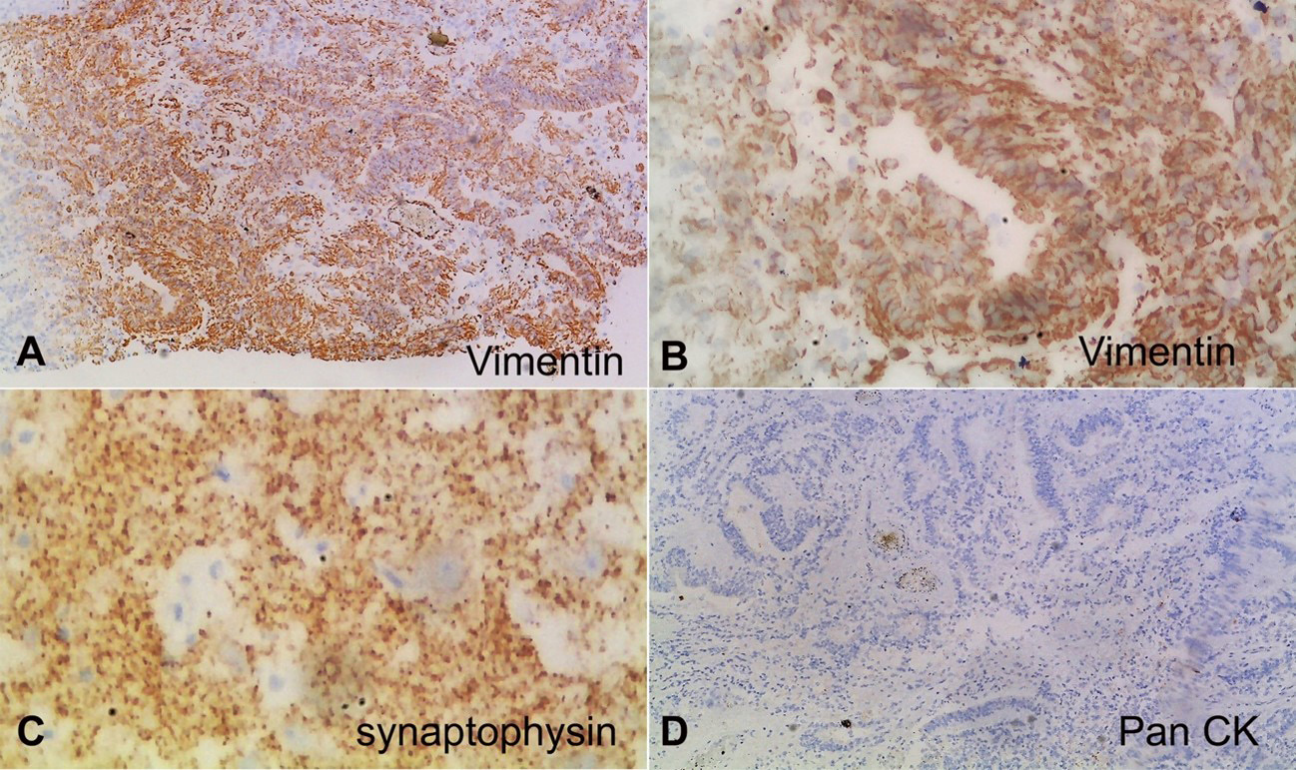
Photomicrographs of the tumor shows– **A** and **B** – diffuse membranous vimentin staining of the multilayered rosettes; **C** – diffuse granular synaptophysin staining of the hypocellular/neuropil zones; **D –** negative staining for pan-cytokeratin (**A, B, C** and **D –** 100X).

**Figure 4:**
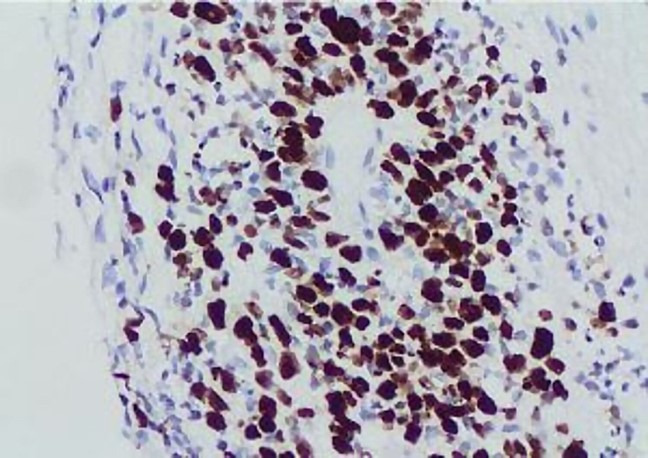
Immunohistochemical reaction for Ki67.

Immunohistochemical stains showed reactivity of the multilayered rosettes for vimentin (Figures 3A &3B) while negative for pan-cytokeratin (Figure 3D). The hypo-cellular areas with abundant neuropil showed reactivity for synaptophysin (Figure 3C). Ki-67 proliferation index was high in the cellular areas (Figure 4).

The presence of multilayered rosettes reactive for vimentin and the abundant neuropil reactive for synaptophysin prompted a diagnosis of embryonal tumor with abundant neuropil and true rosettes (ETANTR) and refined as embryonal tumor with multilayered rosettes, not otherwise specified (WHO Grade 4).

## DISCUSSION

Embryonal tumor with multilayered rosettes (ETMR) is a newly classified central nervous system embryonal tumor, which encompasses a heterogeneous group of highly aggressive malignant tumors. These tumors are composed of primitive cells with distinct morphological and molecular features.^1^ Before the pioneering studies by Korshunov et al.,^3^ the tumors that now include ETMR group were divided into three separate entities on the basis of their histological features. Clinical features and prognosis of these three tumor entities were very similar.

The tumors, termed embryonal tumor with abundant neuropil and true rosettes (ETANTR) are composed of a primitive cell component arranged in sheets and a mature glial, and or neuronal component with an easily appreciable background neuropil. Scattered multilayered rosettes are also an integral component of this tumor.^9^ Tumors termed ependymoblastoma (EBL) are composed of sheets of primitive cells and frequent multilayered (ependymoblastic) rosettes. Medulloepitheliomas (MEPL) are composed of primitive cells arranged in papillae, tubules, and trabeculae with deposition of perioidic acid Schiff (PAS) positive outer membrane at one of the surfaces resembling a primitive neural tube. Multilayered rosettes are also seen in these tumors.^10^^,^^11^ Thus, the presence of a primitive cell population and multilayered rosettes are shared by these tumors.^12^ Li et al.^13^ discovered the unique C19MC amplification in a subset of CNS primitive neuroectodermal tumors (PNETs) with aggressive behavior. Korshunov et al.^3^ performed genetic analysis on 97 such tumors and identified a common genetic alteration, that is, amplification of C19MC locus on chromosome 19 in 93% of cases. This alteration, which was common and specific to these three tumor entities, became their unifying molecular signature. Based on these findings, EBL and ETANTR are now considered two ends of the histologic spectrum, in which EBL represents the undifferentiated and ETANTR the differentiated counterpart.^14^ The 2021 WHO CNS update has redefined and regrouped a significant number of CNS neoplasms by incorporating their molecular characteristics. For ETMR, the presence of C19MC amplification is mandatory for the diagnosis. The diagnosis of ETMR can be made on the presence of C19MC amplification even if multilayered rosettes are not seen. However, when the molecular testing is unavailable, the presence of multilayered rosettes is mandatory and such cases are diagnosed as embryonal tumor with multilayered rosettes, not otherwise specified (NOS).^1^

Most of these tumors are diagnosed at a very young age. 8% of all patients are diagnosed over the age of three years.^3^ Two cases were reported in children over three years of age, one in an eleven-year-old female who presented with complaints of headache and vomiting for one month, MRI showed heterogeneous enhancing lesion with cystic and solid component involving the right frontal lobe extending into the perisylvian region. The solid component was hypointense on T2-weighted (T2W) images, isointense on T1-weighted (T1W) images with minimal contrast enhancement and showed restricted diffusion. The cystic component was hyperintense on T2W images, hypointense on Fluid Attenuation Inversion Recovery (FLAIR) and T1W images without any contrast enhancement.

In this case, the histological features and immunohistochemistry suggested a diagnosis of ETMR, NOS.^15^ The second case was a fifteen-year-old male who presented with headaches and dizziness, brain computed tomography identified a large mass in the left frontotemporal lobe. Histologically, the tumor consisted of typical papillary or trabecular and multilayered rosette pattern resembling MEPL. Immunohistochemically, the tumor cells were positive for LIN28A and Vimentin, but negative for pan-cytokeratin. The FISH analysis showed amplification at the 19q13.42 locus rendering the diagnosis of ETMR, C19MC.^6^ Despite the similarity of the age of these two reported cases with our case, the locations differed, the previous reported cases were supratentorial. Notwithstanding, they can involve any part of the brain but over 70% of them occur in the supratentorial cerebral hemispheres, 30% in the infratentorial region^3^^,^^16^ (brainstem and cerebellum) and rarely in the spine.^17^ Our case presented with an infratentorial tumor (left cerebellum). Most infratentorial tumors were observed to be C19MC negative ETMRs and often reside near the brainstem.^4^ Males and females are equally involved.^4^ Presenting symptoms are similar to other malignant brain tumors that occur at a very young age, and are based on the initial location, the size of tumor and secondary obstruction of cerebrospinal fluid circulation with hydrocephalus. Apart from symptoms of raised intracranial pressure, other symptoms such as paresis, seizures, visual impairment, ataxia and torticollis may occur.^16^^,^^17^ Due to the aggressive nature of the disease, these tumors may acutely present as large tumors with short symptomatic interval leading to a poor pre-and post-operative status with neurological impairment.^18^ ETMRs mostly present as large well and demarcated tumors using magnetic resonance imaging (MRI). The tumors generally show a heterogeneous signal with frequent diffusion restrictions, cystic components, as well as intratumoral hemorrhage.^7^ Compared to other CNS embryonal tumors, the imaging characteristics of ETMRs are similar but the tumor size is overall larger with a mean tumor volume of 115cm^3^ often spanning multiple lobes.^7^^,^^19^

ETMRs show diverse histologic patterns. The diagnosis currently relies heavily on the identification of molecular characteristics by fluorescence in situ hybridization (FISH) or copy number profiling with either SNP arrays, DNA methylation arrays or next generation sequencing (NGS) approaches^4^^,^^20^ ETMRs, regardless of histological variant, react positively for the marker LIN28A with the expression limited to rosette forming cells.^21^ LIN28A expression is rarely seen in other brain tumor entities, which are completely negative or only show some focal expression.^21^^-^23 Other less specific markers useful to the diagnosis of ETMR include the expression of nestin and vimentin in rosette forming cells, which lack expression of neuronal and glial cell markers,^9^^,^^16^ however such markers, for example synaptophysin (SYN), neurofilament protein (NFP), and neuronal nuclei (NeuN)^11^ are expressed in the neuropil zones that may also contain rare populations of cells positive for astrocyte markers such as glial fibrillary acidic protein (GFAP).^1^ Rosette forming cells show negative expression for pan-cytokeratin.6 Our case demonstrated reactivity of the multilayered rosettes for vimentin and neuropil zones for synaptophysin.

Treatment for ETMRs is multimodal. The complete resection is often attempted for patients with localized disease. Surgery of a large tumor of this age group is associated with a high risk for perioperative complications and certain tumor locations preclude complete resection, in particular tumors residing in or near the brainstem.^24^ Due to the rarity of this entity, no specific treatment strategy using chemotherapy has been prospectively evaluated. The current therapeutic strategies are based on prospective trials that enrolled CNS-PNETs and other high risk CNS embryonal tumors, and evaluated the effectiveness of a combined induction chemotherapy and consolidation with high dose chemotherapy followed by autologous stem cell rescue.^25^ These strategies contain different combinations of drugs such as etoposide, cyclophosphamide, vincristine, methotrexate, cisplatin, carboplatin and thiotepa.^8^^,^^25^^,^^26^ Irradiation of ETMR patients is mostly not attempted, since there is no standard treatment protocol for young children that combine craniospinal irradiation with chemotherapy. Preclusion of craniospinal/local irradiation is mainly due to toxicity and associated risk for leptomeningeal spread as observed in medulloblastoma.^27^

Despite intensive and multimodal treatment, the reported outcome is still poor with a five overall survival rates between 0 and 30%. Many patients show aggressive progression of disease, which often is refractory to treatment. A few patients were reported to have been salvaged upon relapse. However, there are also reports of long term survivors.^9^^,^^11^^,^^16^^-^18^,^^28^^-^30

## CONCLUSION

ETMR is a highly aggressive CNS embryonal neoplasm (WHO Grade 4) with extremely poor prognosis. It should be considered in the differential diagnosis of pediatric brain tumors. On histological examination, multilayered rosettes should be carefully searched for and their reactivity for vimentin, and non-reactivity for pan-cytokeratin confirms a diagnosis of ETMR, NOS if immunohistochemistry for LIN28A and genetic testing for amplification of C19MC are unavailable.
